# Structural basis of mevalonate pathway regulation by feedback inhibition of mevalonate kinase

**DOI:** 10.1016/j.jbc.2026.113566

**Published:** 2026-09-17

**Authors:** Rachel L.S. D’Emilia, Kimberly A. McCaskey, Edwin R. Ragwan, Jude H. Kim, Vivian Chang, My M. Tang, Yan Kung

**Affiliations:** Department of Chemistry, Bryn Mawr College, Bryn Mawr, Pennsylvania, USA

**Keywords:** enzyme inhibitor, enzyme structure, inhibitor, isoprenoid, metabolic regulation, mevalonate kinase, mevalonate pathway, protein structure, steroid, X-ray crystallography

## Abstract

The mevalonate pathway diverts acetyl-CoA from central metabolism to produce isopentenyl pyrophosphate and dimethylallyl pyrophosphate, the universal precursors to all steroids and isoprenoids in biology. Mevalonate kinase (MK) catalyzes a key regulatory step of the mevalonate pathway in many eukaryotes, in which several downstream isoprenoid pyrophosphates act as feedback inhibitors. Known MK inhibitors vary widely in size, including isopentenyl pyrophosphate and dimethylallyl pyrophosphate (five carbons, C_5_), geranyl pyrophosphate (C_10_), farnesyl pyrophosphate (C_15_), and geranylgeranyl pyrophosphate (C_20_). These inhibitors compete for binding at a single site, yet how the enzyme is able to accommodate inhibitors of such varying sizes remains unknown. In this work, we first characterized the inhibition kinetics of all five of these inhibitors using MK from *Saccharomyces cerevisiae* (ScMK), revealing an inhibition potency that ranges approximately two orders of magnitude. We then solved X-ray crystal structures of ScMK bound to all five inhibitors as well as the structure of apo ScMK to shed light on how inhibitors of such varying sizes may all bind to the same site. In all, this suite of inhibitor-bound MK structures provides the first comprehensive structural depiction of MK feedback inhibition, a key regulatory mechanism of the mevalonate pathway of steroid and isoprenoid precursor biosynthesis.

Found in all domains of life, the mevalonate pathway is one of two biological routes to isopentenyl pyrophosphate (IPP) and dimethylallyl pyrophosphate (DMAPP) (Fig. 1), which are the universal five-carbon (C_5_) building blocks required for the biosynthesis of all steroids and isoprenoids (terpenes/terpenoids), the largest and most structurally diverse class of natural products. DMAPP is sequentially condensed with one or more equivalents of IPP by prenyltransferases to produce geranyl pyrophosphate (GPP) (C_10_), farnesyl pyrophosphate (FPP) (C_15_), geranylgeranyl pyrophosphate (GGPP) (C_20_), and so on. These isoprenoid pyrophosphate intermediates may then be converted by terpene synthases/cyclases to their final isoprenoid products, which include monoterpenes (C_10_), sesquiterpenes (C_15_), and diterpenes (C_20_). Alternatively, two equivalents of FPP may be condensed by squalene synthase to form squalene, the triterpene precursor to all steroids in biology.

**Figure 1:**
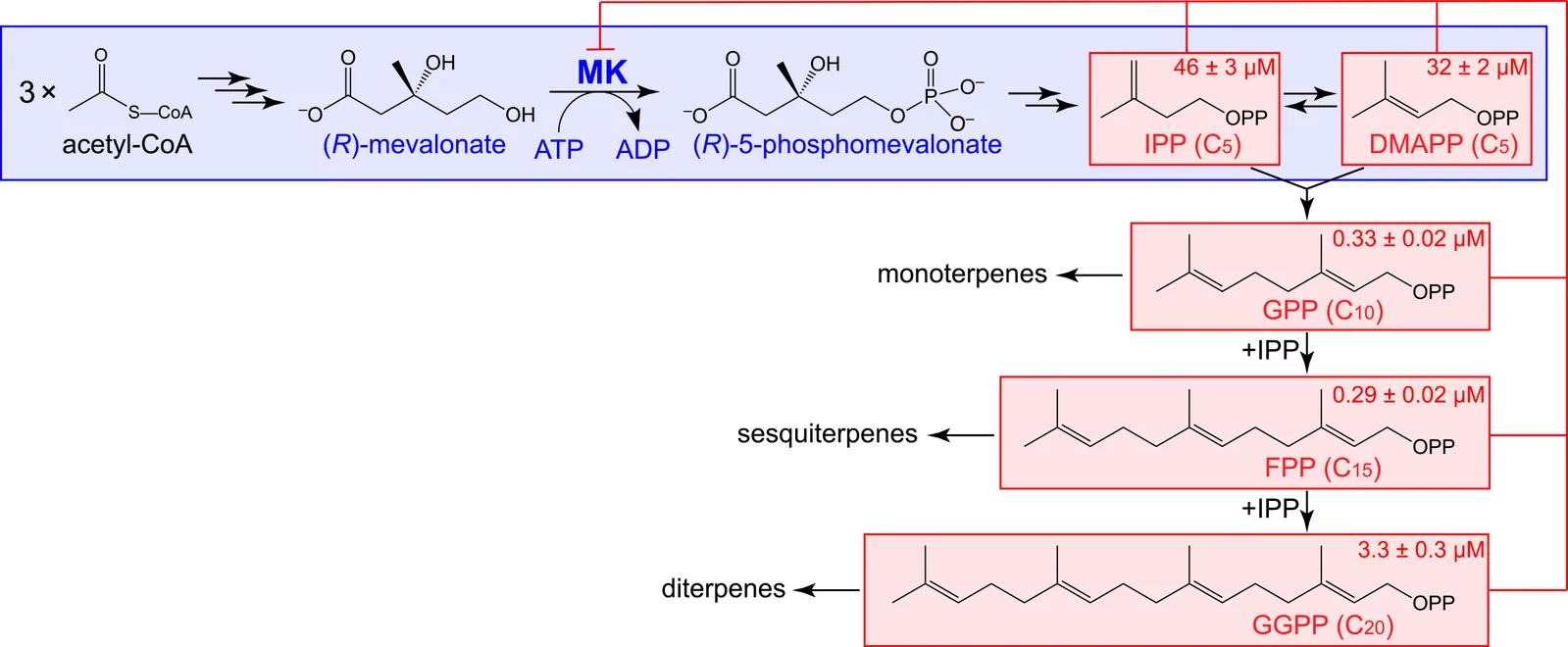
**Mevalonate pathway and downstream metabolites.** The mevalonate pathway (*blue box*) is composed of seven enzymatic steps, including the ATP-dependent phosphorylation of (*R*)-mevalonate by MK *(blue text)*. The five-carbon (C_5_) isomeric products of the pathway, IPP and DMAPP, may then be condensed to form GPP (C_10_), FPP (C_15_), and GGPP (C_20_) by dedicated prenyltransferases. All five of these products can act as feedback inhibitors of MK activity (*red text*, *boxes*, *lines*), with corresponding *K*_i_ values shown. GPP, FPP, and GGPP are used as precursors to monoterpenes, steroids and sesquiterpenes, and diterpenes, respectively. “P” represents a phosphate group. DMAPP, dimethylallyl pyrophosphate; FPP, farnesyl pyrophosphate; IPP, isopentenyl pyrophosphate; GGPP, geranylgeranyl pyrophosphate; GPP, geranyl pyrophosphate; MK, mevalonate kinase.

Given its critical role in metabolism and biosynthesis, the mevalonate pathway and isoprenoid precursor biosynthesis are prominent targets in both human medicine and in synthetic biology and metabolic engineering. For example, the blockbuster class of statin drugs targets the mevalonate pathway enzyme 3-hydroxy-3-methylglutaryl reductase to inhibit sterol biosynthesis, thereby reducing blood cholesterol levels in people with hypercholesterolemia. Nitrogen-containing bisphosphonates are the most commonly prescribed drugs to treat osteoporosis and target the prenyltransferase FPP synthase, hindering protein prenylation in osteoclasts. Interestingly, many different types of cancer cells possess strongly elevated mevalonate pathway activity, suggesting that downregulating the mevalonate pathway is a promising approach for anticancer treatment (1, 2, 3, 4, 5). The mevalonate pathway is also used in synthetic biology and metabolic engineering, where microorganisms have been engineered to produce isoprenoid derivatives as diverse as anticancer drugs (6, 7, 8, 9), antimalarial drugs (10, 11), cannabinoids (12, 13, 14), biofuels (15, 16, 17, 18, 19, 20), and other commodity chemicals (21, 22, 23) through the mevalonate pathway. Therefore, greater insight into mevalonate pathway biochemistry and regulation is of immense interest in both human health and in industry.

Mevalonate kinase (MK) catalyzes the first of three consecutive ATP-consuming steps of the mevalonate pathway, the phosphorylation of mevalonate to 5-phosphomevalonate. MK has evolved to be a key point of mevalonate pathway regulation in many organisms (24, 25, 26, 27, 28, 29, 30, 31, 32, 33), where MK enzymatic activity is inhibited by several downstream metabolites. Known endogenous MK inhibitors comprise the products of the mevalonate pathway, IPP and DMAPP, as well as the larger downstream isoprenoid pyrophosphate intermediates GPP, FPP, and GGPP (Fig. 1). Recent studies on *Homo sapiens* MK (HsMK) suggest that isoprenoid monophosphates can also serve as potent inhibitors (34). Prior work has established a competitive mode of inhibition where feedback inhibitors all bind to the ATP-binding site of the enzyme (24, 25, 27, 28, 32, 35), but how MK is able to accommodate so many different inhibitors of such varying sizes at a single site that must also bind the ATP substrate is unknown.

Crystal structures of MK from several different organisms have been solved, including apo structures (32, 35, 36, 37, 38, 39, 40, 41), as well as structures bound with substrate mevalonate (32, 39, 42) and product 5-phosphomevalonate (42). However, to date only one structure of any MK has been determined bound with ATP (43), which was found within a deep surface cleft of MK from *Rattus norvegicus* (RnMK) (Fig. 2). Interestingly, another structure of RnMK was determined where the enzyme is bound to a synthetic analog of FPP, farnesyl thiophosphate (FSP) (35). Consistent with competitive inhibition, FSP occupies the ATP-binding site such that its single phosphate group binds Mg^2+^, taking the place of the β-phosphate of ATP, while its C_15_ alkyl chain extends into the same region that binds the adenosine of ATP (Fig. 2). However, until now no structure of MK bound with any endogenous feedback inhibitor has been determined.

**Figure 2:**
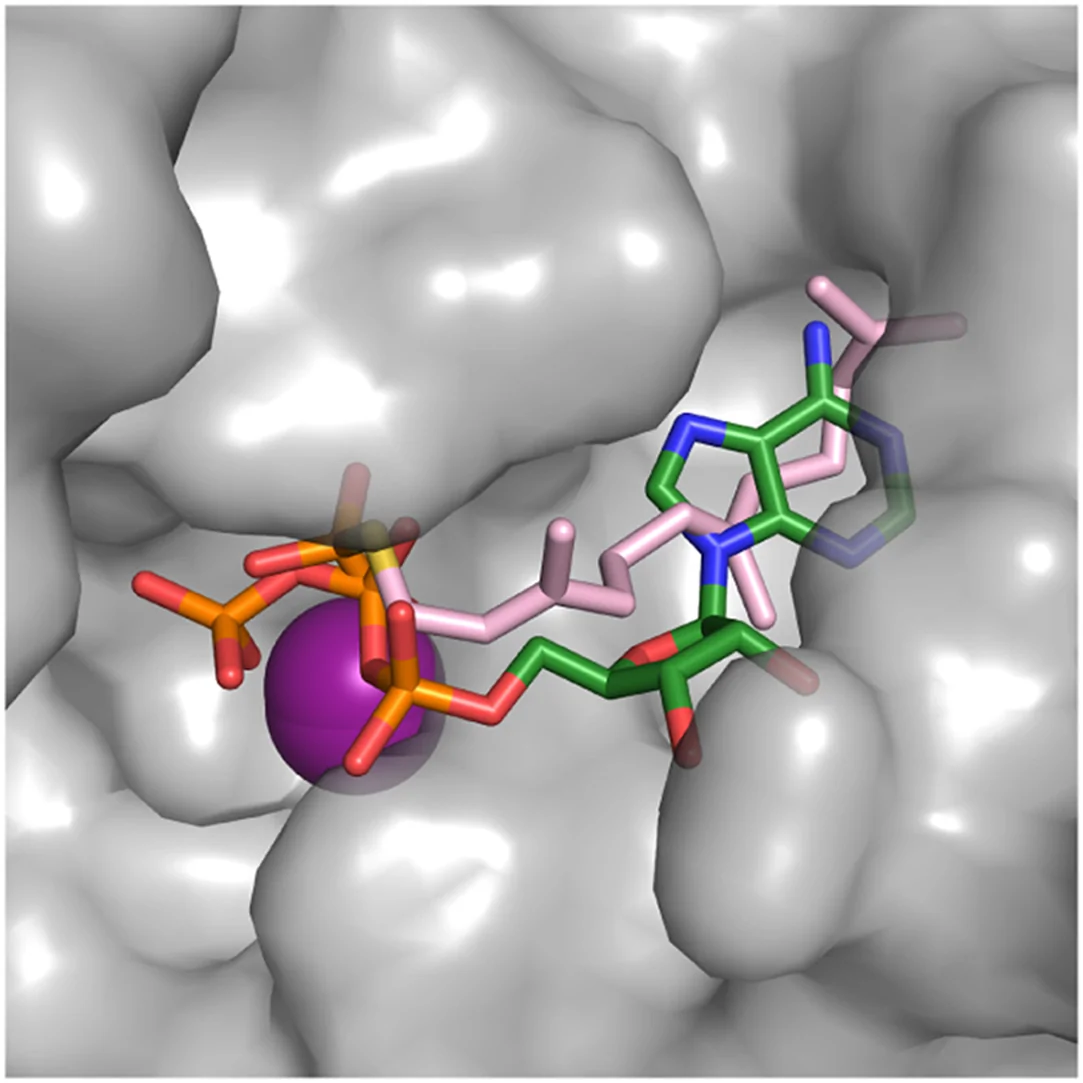
**ATP-binding site of MK.** Previously determined crystal structures of RnMK (semitransparent *gray surface*) bound with either ATP (*green*, PDB entry 1KVK) (43) or with FSP, *pink*, PDB entry 2R42), a synthetic analog of the endogenous inhibitor FPP (35). Both ATP and FSP coordinate Mg^2+^ (*violet sphere*) and bind the same site, indicative of competitive inhibition. FSP, farnesyl thiophosphate; MK, mevalonate kinase; RnMK, *Rattus norvegicus* mevalonate kinase.

In this work, we first characterized the inhibition kinetics of MK from *Saccharomyces cerevisiae* (ScMK) in the presence of IPP, DMAPP, GPP, FPP, and GGPP. We then solved crystal structures of ScMK bound to all of these inhibitors, in addition to the structure of apo ScMK, revealing how such a wide range of inhibitor sizes, from C_5_- to C_20_-pyrophosphates, are all able bind at the ATP-binding site of the enzyme. As the mevalonate pathway is responsible for the biosynthesis of countless steroid and isoprenoid derivatives in biology, this work opens the door to the development of drugs that target MK for inhibition, either in humans or in pathogenic organisms, to reduce metabolic flux toward steroid and isoprenoid biosynthesis. In addition, these crystal structures also represent the first structures of ScMK, the primary workhorse used in metabolic engineering for the industrial production of isoprenoids and their derivatives through the mevalonate pathway. Therefore, the work presented here also sets the stage for protein engineering efforts that may attenuate or overcome intracellular MK feedback inhibition, as MK activity is a known metabolic bottleneck that can limit product yields in organisms engineered to produce isoprenoids (44, 45, 46, 47).

## Results

### Inhibition kinetics

Prior published ScMK studies reported inhibition dissociation constants, *K*_i_, for only DMAPP, GPP, and FPP (27). Therefore, we performed steady-state inhibition kinetics (Figs. S1 and S2) using all five inhibitors—IPP, DMAPP, GPP, FPP, and GGPP—yielding the *K*_i_ values shown in Figure 1 and Table 1. As a comparison, the *K*_i_ values determined here for DMAPP, GPP, and FPP of 32 ± 2 μM, 0.33 ± 0.02 μM, and 0.29 ± 0.02 μM, respectively, are highly similar to the previously reported *K*_i_ values of 34 ± 17 μM, 0.25 ± 0.09 μM, and 0.13 ± 0.08 μM, respectively (27). We also determined the *K*_m_ of ScMK with respect to ATP to be 88.4 ± 5.9 μM (Fig. S1), which is significantly lower than the previously reported value of 650 ± 72 μM (27), suggesting tighter ATP binding with our ScMK sample. This discrepancy could be partially attributed to a difference in construct, though our *K*_m_ value is also very similar to those reported for HsMK of 74 ± 9 μM (25) and 57 ± 4 μM (29). We also determined a turnover number, *k*_cat_, of 10.2 ± 0.2 s^–1^, which is comparatively more similar to the previously reported value for ScMK of 38 ± 5 s^–1^ (27).

**Table 1:** ScMK inhibitor dissociation constants, *K*_i_

Inhibitor	C_n_	*K*_i_ (μM)
IPP	5	46 ± 3
DMAPP	5	32 ± 2
GPP	10	0.33 ± 0.02
FPP	15	0.29 ± 0.02
GGPP	20	3.3 ± 0.3

Number of carbon atoms, C_n_.

Interestingly, *K*_i_ values for all five ScMK inhibitors span two orders of magnitude. The smallest inhibitors exhibit the weakest inhibition potency, with *K*_i_ values for IPP and DMAPP of 46 ± 3 μM and 32 ± 2 μM, respectively. Binding ScMK stronger by approximately one order of magnitude is the largest inhibitor, GGPP, with *K*_i_ of 3.3 ± 0.3 μM. The strongest inhibition potency is afforded by intermediate-sized GPP and FPP, with *K*_i_ values one order of magnitude lower still, at 0.33 ± 0.02 μM and 0.29 ± 0.02 μM, respectively. These results suggest a “Goldilocks” zone for inhibitor binding: the smallest inhibitors (C_5_) have limited interaction surfaces available for binding and are the weakest, while the largest inhibitor (C_20_) may be too sterically large and bulky to be easily accommodated in the binding pocket, leaving medium-sized inhibitors (C_10–15_) to be “just right” and the most potent. Notably, ScMK appears to bind these inhibitors as tightly to significantly more tightly than its ATP substrate.

### Overall ScMK structure

To shed light on the structural basis of MK inhibition, including the ability to bind a wide range of inhibitor sizes, we solved crystal structures of ScMK with and without all five inhibitors bound (Table S1). Prior structural work indicated that MK exists as a homodimer (32, 35, 36, 39, 41, 42, 43) and is consistent with solution studies (25, 26), although one MK homolog crystallized as a monomer (37, 38), and MK from the parasite *Trypanosoma evansi* was found to be a homotetramer in solution (48). In all homodimeric MK structures, protomers are arranged side-by-side with two-fold symmetry such that the active sites are located on opposite faces of the homodimer. ScMK adopts the same homodimeric structure (Fig. 3), where one protomer is present in the asymmetric unit and the second protomer is generated by crystallographic symmetry. The ScMK homodimer aligns well with all previously determined structures of homodimeric eukaryotic MK homologs, with root-mean-square deviation for C_α_ atoms (rmsd) values of 0.85 to 1.09 Å among ScMK, RnMK, and HsMK (Fig. 3).

**Figure 3:**
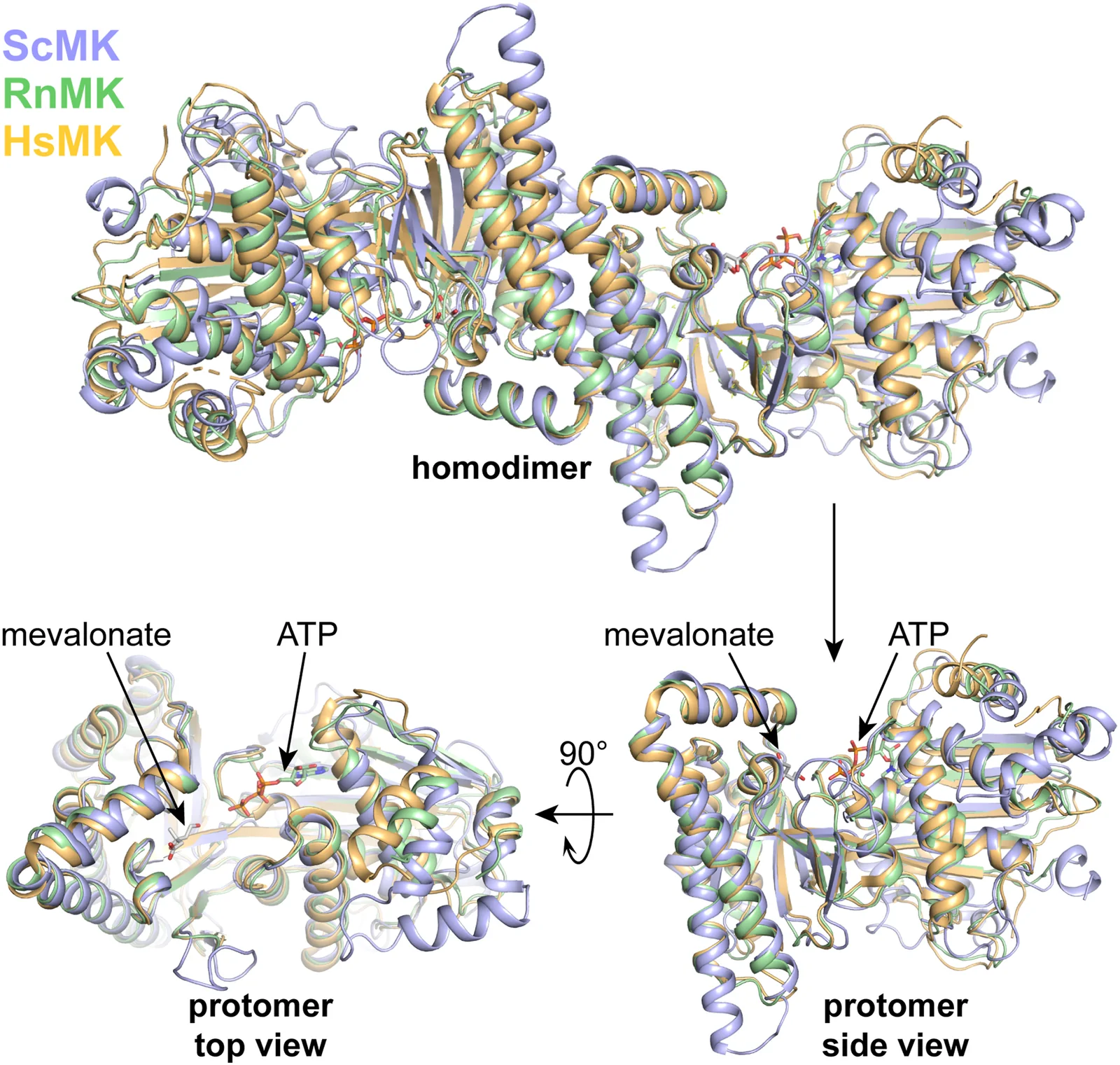
**Overall homodimeric structure of MK.** The crystal structure of homodimeric apo ScMK presented here in *light blue* is aligned with prior structures of ATP-bound RnMK (43) in *light green* (PDB entry 1KVK) and apo HsMK (43) in *light orange* (PDB entry 2R3V), with root-mean-square deviation for C_α_ atoms (rmsd) of 0.85 Å and 1.09 Å, respectively. Structures were aligned together using one protomer (*right*) of each homodimer. Mevalonate binding (*gray sticks*) based on a structural alignment of the apo ScMK structure and the prior structure of mevalonate-bound MK from *Methanosarcina mazei* (PDB entry 6MDE) (42), with rmsd of 1.06 Å. The ScMK asymmetric unit contains one protomer, with the homodimer completed by crystallography symmetry. FPP, farnesyl pyrophosphate; HsMK, *Homo sapiens* mevalonate kinase; MK, mevalonate kinase; RnMK, *Rattus norvegicus* mevalonate kinase; ScMK, *Saccharomyces cerevisiae* mevalonate kinase.

The overall structure of each MK protomer can be described as having two domains, with the active site residing in a deep interdomain surface cleft (Fig. 3). Here, ATP binds the N-terminal domain with its phosphate groups pointing toward the C-terminal domain (43), while mevalonate binds within a cavity between the N- and C-terminal domains (39, 42). A conserved His19 residue (ScMK numbering) is involved in mevalonate binding and is believed to be involved in selecting the correct (*R*)-enantiomer of the substrate (39).

All six structures of ScMK presented here align very well with each other and are virtually superimposable overall, with rmsd values of 0.10 to 0.18 Å, as would be expected given that all structures were obtained from apo ScMK crystals. The primary differences between the structures are the positions of two loops—Phe54–Phe59 and Ser107–His111—which also exhibited a somewhat greater degree of disorder than the rest of the protein, though backbone density in these regions was nonetheless continuous in all structures (Fig. S3). Notably, residues of both loops are also near the ATP- and inhibitor-binding site and are discussed in greater detail below.

### Inhibitor binding

To obtain the five inhibitor-bound structures of ScMK, crystals of the apo protein were soaked in solutions that contained 5 mM IPP, DMAPP, GPP, FPP, or GGPP for 30 to 45 min before flash-freezing in liquid nitrogen for X-ray data collection. Upon structure determination, well-defined positive *mF*_o_–*d**F*_c_ difference electron density was present for each corresponding inhibitor in the ATP-binding site (Fig. 4). Upon refinement, the pyrophosphate head groups of all inhibitors occupy a similar position and coordinate Mg^2+^ (Fig. 5), with conserved Ser149 and a water molecule serving as additional ligands to the Mg^2+^ ion. The side chain of His195 also points toward Mg^2+^ in all inhibitor-bound structures, but due to the slightly elevated distances of 3.0 to 3.4 Å across these structures and an uncommonly observed nitrogen coordination to Mg^2+^ it is unclear whether His195 is a direct ligand of the Mg^2+^ ion.

**Figure 4:**
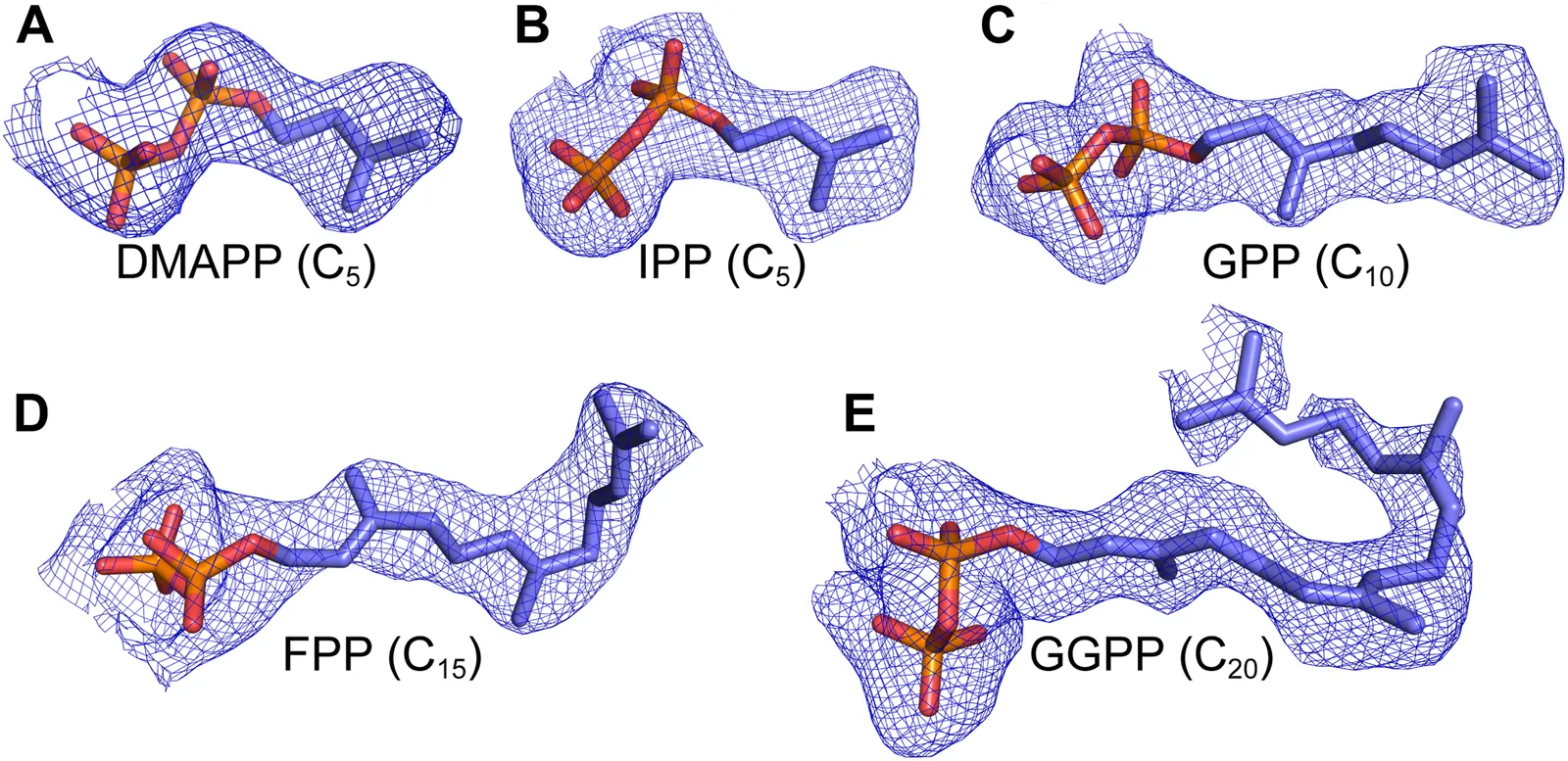
**Electron density of ScMK inhibitor binding sites.** Polder *mF*_o_–*d**F*_c_ omit density calculated in Phenix (55) for *A*, IPP; *B*, DMAPP; *C*, GPP; *D*, FPP; and *E*, GGPP in *blue mesh* contoured at 3.5 σ. DMAPP, dimethylallyl pyrophosphate; FPP, farnesyl pyrophosphate; GGPP, geranylgeranyl pyrophosphate; GPP, geranyl pyrophosphate; IPP, isopentenyl pyrophosphate; ScMK, *Saccharomyces cerevisiae* mevalonate kinase.

**Figure 5:**
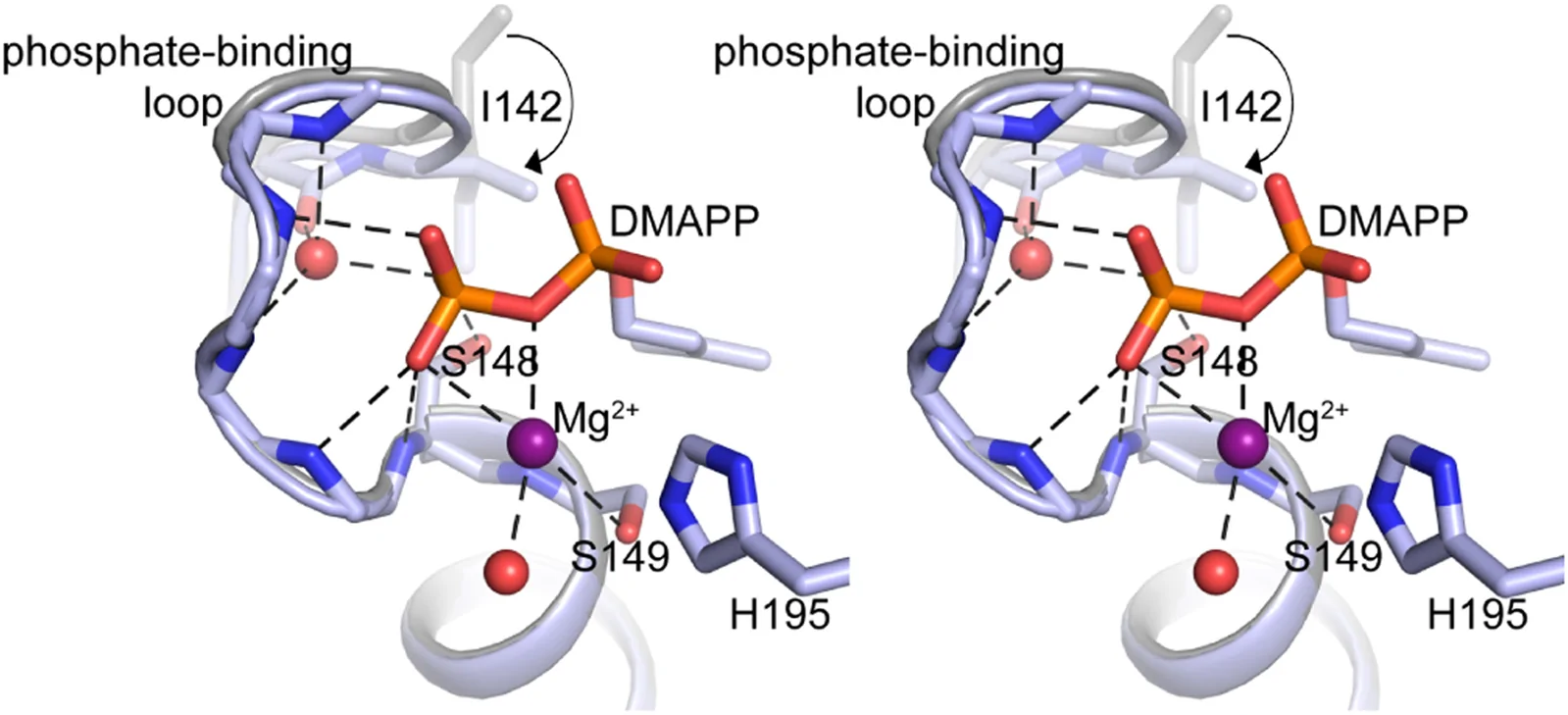
**Phosphate-binding region of ScMK.** Stereoview of the phosphate-binding site in structure of ScMK (*light blue*) bound with DMAPP (*sticks*), which is the highest-resolution (1.93 Å) structure of inhibitor-bound ScMK presented here. Mg^2+^ and water molecules are shown as a *violet* and *red spheres*, respectively. The apo ScMK structure is shown in *gray*. *Dashed lines* represent hydrogen bonds. DMAPP, dimethylallyl pyrophosphate; ScMK, *Saccharomyces cerevisiae* mevalonate kinase.

The phosphate-binding region in these ScMK structures is similar to those observed in the prior ATP- and FSP-bound RnMK structures and can be described as having a foundation and a loop that together cradle the phosphate groups (Fig. 5). The foundation is formed by Mg^2+^ and the N-terminal end of an α-helix, which allows favorable interactions between the negatively charged phosphates and the δ-positive character at the N-terminus of the helix. The first residue of this helix is Ser148, which forms a hydrogen bond with the β-phosphate groups of the inhibitors. Folded over the pyrophosphate is a previously described phosphate-binding loop that precedes the foundation helix in the sequence and forms a PXGXGLGSSA motif (43). In ScMK, this phosphate-binding loop includes P141, I142, Gly143, Ala144, Gly145, Leu146, Gly147, and the aforementioned Ser148; interestingly, one backbone carbonyl and six backbone NH groups of this loop all form hydrogen bonds with either the pyrophosphate directly or a single water molecule that itself also forms a hydrogen bond with the pyrophosphate. Compared to the apo ScMK structure, this loop moves only very slightly toward the phosphate groups upon inhibitor binding, with Ile142 of the loop changing conformation to clamp down over the inhibitor and cover the inhibitor-binding site (Fig. 5).

From the phosphates, the alkyl groups of the inhibitors emanate away from the Mg^2+^ site and sit in the same general region as the adenosine of ATP in the ATP-bound RnMK structure (43). Starting with the smallest inhibitors IPP and DMAPP, their five-carbon nonpolar hydrocarbon chains occupy an initial level of a hydrophobic pocket that is composed of Phe110, Ala114, Ile142, I152, Ile194, and Phe54 (Fig. 6*A*). In these two structures as well as the structure of apo ScMK, electron density for the side chain of Phe54 is broad and split into two distinct orientations (Fig. S4*A*). Correspondingly, the best-fit refinement contains two alternate conformations for Phe54, one of which blocks larger inhibitors from binding in this hydrophobic pocket (Figs. 6*A*, S3*C*, and S4*A*).

**Figure 6:**
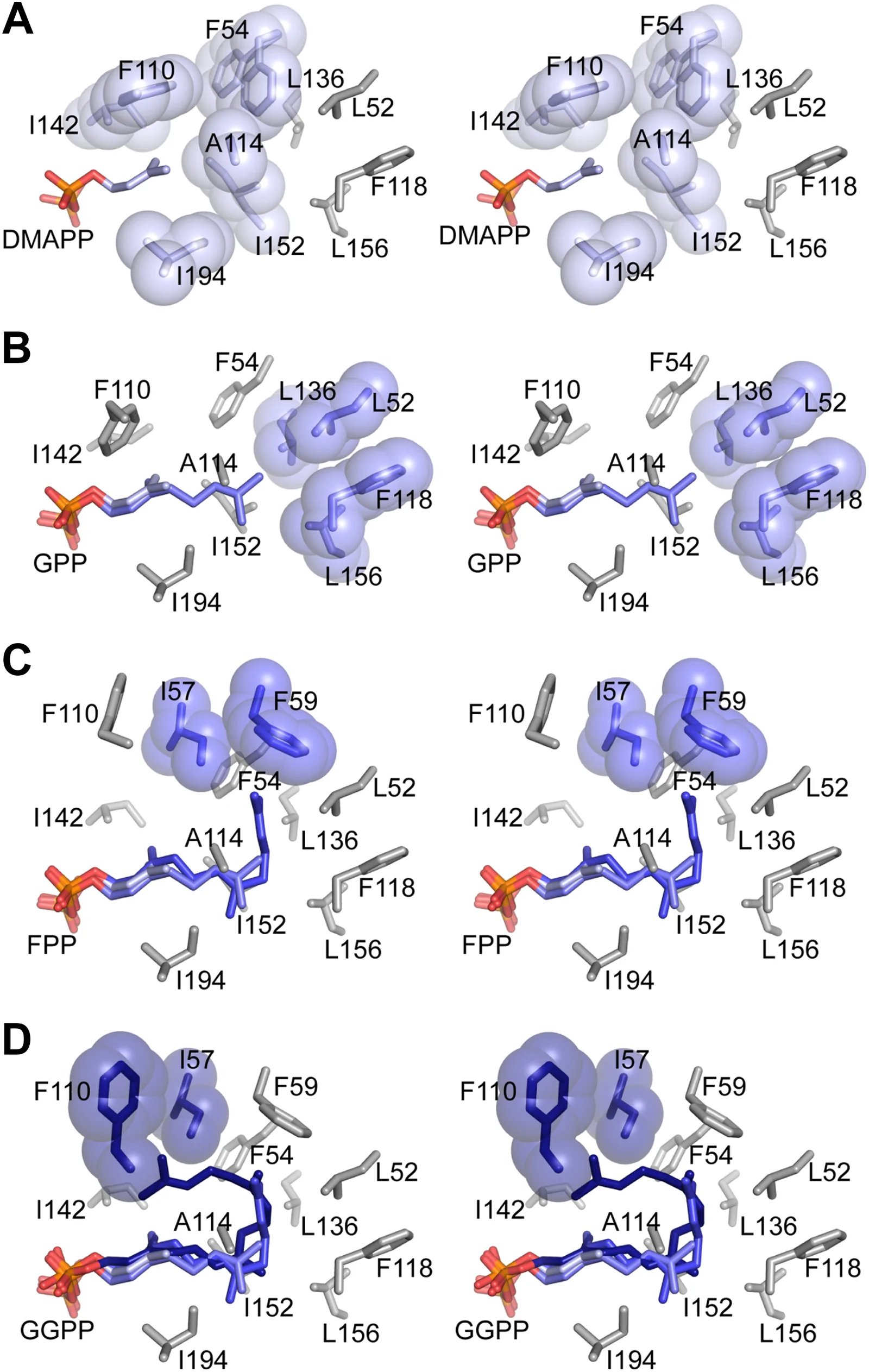
**Stereoviews of the inhibitor binding site of ScMK.***A*, ScMK bound with DMAPP (carbon atoms in *pale blue*), with residues that form the hydrophobic inhibitor-binding pocket in *pale blue sticks* and spheres. An alternate conformation of Phe54 blocks off the hydrophobic pocket from a deeper level of hydrophobic residues (*gray sticks*). *B*, ScMK bound with GPP (carbon atoms in *light blue*), with residues that form the deeper level of the hydrophobic pocket in *light blue sticks and spheres*. DMAPP from *A* is shown in pale *blue sticks*, and other hydrophobic residues are shown in *gray sticks*. *C*, ScMK bound with FPP (carbon atoms in *blue*), with additional hydrophobic residues that interact with the tail of FPP shown in *blue sticks and spheres*. DMAPP from *A*, GPP from *B*, and other hydrophobic residues are shown in *pale blue*, *light blue*, *and gray sticks*, respectively. *D*, ScMK bound with GGPP (carbon atoms in *dark blue*), with residues that interact with the tail of GGPP shown in *dark blue sticks* and *spheres*. DMAPP from *A*, GPP from *B*, FPP from *C*, and other hydrophobic residues are shown in *pale blue*, *light blue*, *blue*, *and gray sticks*, respectively. DMAPP, dimethylallyl pyrophosphate; FPP, farnesyl pyrophosphate; GGPP, geranylgeranyl pyrophosphate; GPP, geranyl pyrophosphate; ScMK, *Saccharomyces cerevisiae* mevalonate kinase.

As the inhibitor chain lengthens by five carbons from IPP/DMAPP to GPP, Phe54 adopts a single conformation that no longer blocks larger inhibitors (Fig. S4*B* and 6*B*), as it moves out of the way to grant access to a deeper level of the hydrophobic pocket composed of Leu52, Phe118, Leu136, and Leu156, which interact with the five additional carbons of GPP (Figs. 6*B* and S3*C*). These residues form the back wall of this enlarged hydrophobic pocket; therefore, when the inhibitor lengthens further to FPP, the hydrophobic wall forces the additional carbons of the inhibitor to make a ∼90° turn up the wall to also interact with hydrophobic residues Ile57 and Phe59 (Fig. 6*C*). Notably, FPP now extends directly into the location that was occupied by the blocking alternate Phe54 conformation in the apo, IPP-, and DMAPP-bound structures.

Finally, when the alkyl chain lengthens even further to GGPP, Phe59 sterically blocks a linear continuation of the chain, causing the additional five carbons of GGPP to turn a further ∼90° such that the chain has doubled back onto itself (Fig. 6*D*). Therefore, GGPP overall adopts a hook shape that resembles the letter “J” with a hairpin ∼180° curve. Its terminal five carbons now extend into the location that was previously occupied by Phe110, which has now rotated ∼180° around to move out of the way (Figs. 6*D* and S3*D*). This rotation also causes the loop that contains Phe110 to lift away slightly from the inhibitor (Fig. S3). Here, the terminal five carbons of GGPP are wedged between Ile57, the new Phe110 conformation, and the other carbon atoms of its chain.

Taken together, these structures depict all inhibitors occupying the same hydrophobic region within the ATP-binding site of MK, with larger inhibitors curling up to adopt a compact size in order to fit within the binding pocket. Only small loop and side chain movements are necessary to enable the enzyme to accommodate and adapt to inhibitors of varying sizes.

## Discussion

The structural biochemistry of the mevalonate pathway is of enormous interest, as it is currently used as both a druggable target for the treatment of a wide variety of human diseases as well as a valuable pipeline for the biological production of isoprenoid-derived commodity chemicals. MK lies at a key regulatory point of the mevalonate pathway, as its activity is controlled by feedback inhibition in many organisms. The crystal structures of ScMK presented here offer a distinct view of MK structure, as ScMK adopts a similar overall fold yet shares fairly low sequence identity with the other eukaryotic MK homologs whose structures are known: 27%, 31%, 31%, and 33% identity with MKs from *Leishmania major*, *Ramazzottius varieornatus* (RvMK), RnMK, and HsMK, respectively. Furthermore, these structures are also of value to metabolic engineers and synthetic biologists, as ScMK is precisely the MK homolog used in countless microbial strains that have been engineered to produce isoprenoid derivatives using the mevalonate pathway specifically from *S. cerevisiae*, yet no structure of this MK has been determined until now. Therefore, the suite of inhibitor-bound ScMK structures presented here may open the door for enzyme engineering efforts to attenuate or overcome MK feedback inhibition, an intrinsic regulatory mechanism that can limit product yields. Structural insight into MK inhibition also has the potential to motivate the development of new drugs that target the mevalonate pathway in both humans and in pathogenic organisms; notably, the structures of ScMK here are also the first structures reported of a fungal MK.

A comparison between the apo ScMK structure and the inhibitor-bound structures reveals very few differences. All six structures are virtually superimposable, indicating that ligand binding to the ATP/inhibitor-binding site does not involve major structural changes, nor does the binding of small versus large inhibitors. As described above, there are just two loops near the ATP/inhibitor-binding site that move slightly upon inhibitor binding (Fig. S3). The first loop is composed of Phe54–Phe59, with Phe54 in particular changing from being relatively disordered, flexible, and adopting two primary conformations in the apo, IPP-, and DMAPP-bound structures to adopting a single ordered position that is out of the way when binding larger inhibitors GPP, FPP, and GGPP, as described above (Figs. 6 and S3). A sequence alignment (Fig. 7) shows that this position is also occupied by a bulky hydrophobic residue (Phe, Leu, or Met) in all eukaryotic MKs that are known to exhibit feedback inhibition. The second flexible loop is composed of Ser107–His111 and contains Phe110, which as described above changes its conformation to accommodate inhibitors of different sizes and thereby alters the positioning of this loop (Figs. 6 and S3). Interestingly, this residue is not conserved (Fig. 7), which might reflect a unique role for this position as detailed below. Further, even in the absence of inhibitors both loops in the apo structure exhibit a somewhat elevated degree of disorder compared to the rest of the structure, with an average B-factor of 67.2 Å^2^ compared to the average for the entire protein of 52.6 Å^2^. This loop is also disordered in prior structures of FSP-bound RnMK (35) and mevalonate-bound RvMK (32). Taken together, these data all indicate that these two loops have a degree of inherent flexibility that enables ligand binding and helps to accommodate varying inhibitor sizes.

**Figure 7:**
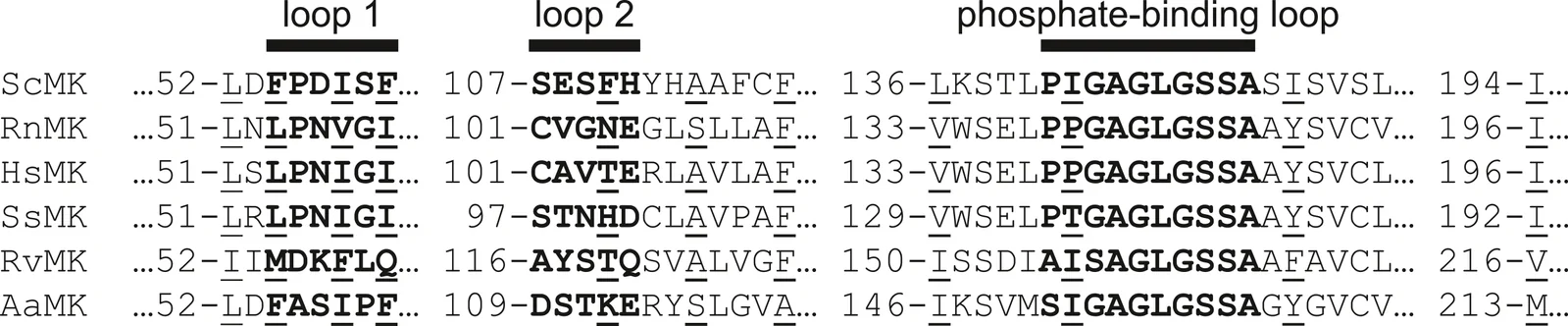
**Sequence alignment of all eukaryotic MK homologs known to exhibit feedback inhibition**, which are ScMK, RnMK, HsMK, MK from *Sus scrofa* isoform 1 (SsMK), RvMK, and MK from *Aedes aegypti* (AaMK). Flexible loops 1 and 2 as well as the phosphate-binding loop are shown in *bold*. Hydrophobic residues described in the text are underlined. AaMK, *Aedes aegypti* mevalonate kinase; HsMK, *Homo sapiens* mevalonate kinase; MK, mevalonate kinase; RvMK, *Ramazzottius varieornatus* mevalonate kinase; RnMK, *Rattus norvegicus* mevalonate kinase; ScMK, *Saccharomyces cerevisiae* mevalonate kinase.

The kinetics work reported here reveals a hierarchy of inhibition potencies reflected in values of the inhibition dissociation constant, *K*_i_, that span roughly two orders of magnitude. The strongest inhibitors, GPP and FPP, are intermediate in size with 10 and 15 carbon atoms, respectively. Our crystal structures of ScMK bound to these inhibitors show that they are accommodated within a well-ordered hydrophobic pocket lined with many nonpolar side chains that make extensive hydrophobic interactions with the inhibitors (Fig. 6, *B* and *C*). These many interactions likely contribute to the strong inhibition potencies observed, as these inhibitors are large enough to take advantage of a deep and extensive hydrophobic pocket. However, the weakest inhibitors, IPP and DMAPP, are the smallest in size and have only five carbon atoms. With only half or one-third as many carbon atoms as GPP or FPP, respectively, our structures of ScMK bound to these inhibitors illustrate more limited hydrophobic interactions with fewer nonpolar side chains (Fig. 6*A*), which rationalize the weaker binding observed. As described above, Phe54 may play a door-like role that “opens” to allow inhibitors larger than IPP and DMAPP access to a deeper hydrophobic region for greater nonpolar interactions with additional hydrophobic side chains, which enables tighter binding and greater inhibition potency.

Between these extremes, the largest inhibitor, GGPP, contains 20 carbon atoms and exhibits an intermediate inhibition potency: stronger than IPP and DMAPP by about one order of magnitude in *K*_i_, but also weaker than GPP and FPP by about one order of magnitude. As mentioned above, only subtle changes in side chain and loop conformation are needed to accommodate GGPP in this site, with the same residues making hydrophobic interactions with GGPP as with FPP (Fig. 6*D*), even as GGPP is the weaker inhibitor. Therefore, it is possible that weaker binding may instead be attributed to a penalty associated with the need for the large GGPP molecule to adopt a more compact conformation for binding. Indeed, our GGPP-bound structure shows that as the largest and bulkiest inhibitor, it must curl up into a “J” shape in order to fit into the same hydrophobic pocket (Fig. 6*D*). This suggests how additional factors, other than the hydrophobic interactions observable in crystal structures, may also contribute to the overall differences in inhibition reflected in steady-state kinetic values. These factors include the likelihood of decreased ligand conformational entropy and greater desolvation that would contribute to greater energetic penalties for GGPP binding.

As all inhibitors occupy the ATP-binding site, it is especially useful to compare these inhibitor-bound ScMK structures to the previously determined ATP- and FSP-bound RnMK structures (35, 43), the only currently available MK structures with any ligands bound in the ATP-binding site. Starting with the smallest inhibitors, IPP and DMAPP, their nonpolar alkyl chains occupy the same region in ScMK as the ribose ring of ATP in RnMK (Fig. 8*A*). When ATP is bound, the 2′- and 3′-hydroxyl groups of ribose make hydrogen bonds with Ser108 and the backbone carbonyl of Asn104 (RnMK numbering), respectively; the side chain of Asn104 is turned away from ATP and faces the bulk solvent exterior (Fig. 8*A*, green). Naturally, these two hydrogen bonds cannot form when nonpolar inhibitors are bound instead of ATP. Indeed, in the structure of FSP-bound RnMK the hydrocarbon chain of FSP is bound in a position that is raised higher in the binding pocket, slightly above the ribose of ATP and thus more distant from Ser108 and Asn104 (Fig. 8*A*, pink). This difference in ligand positioning prevents a polar/nonpolar clash and suggests a strategy for how RnMK binds both ATP and nonpolar inhibitors in the same site.

**Figure 8:**
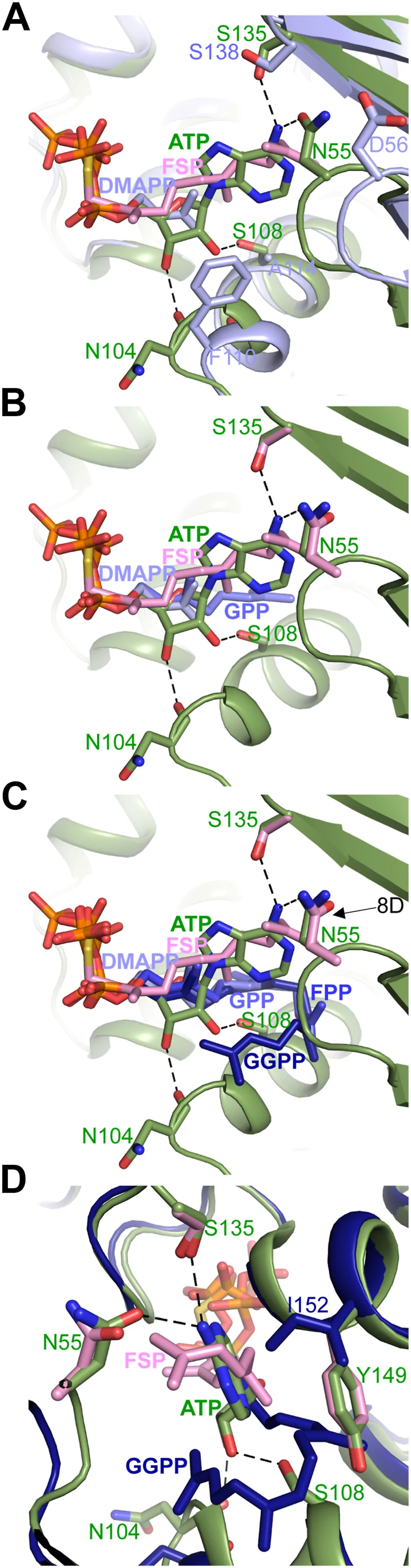
**Structural comparisons of MK bound to ATP and inhibitors.***A*, aligned structures of DMAPP-bound ScMK in *pale blue* and ATP-bound RnMK in *green*, with FSP from the aligned FSP-bound RnMK structure in *pink sticks*. *B*, structure of ATP-bound RnMK in *green*, with FSP (*pink sticks*), DMAPP (*pale blue sticks*), and GPP (*light blue sticks*) from corresponding aligned structures of inhibitor-bound MK. *C*, Same as *B*, plus FPP (*slate blue sticks*) and GGPP (*dark blue sticks*) from corresponding aligned structures of inhibitor-bound ScMK. *D*, side-view of *C*, as indicated by the *arrow in C*, with GGPP-bound ScMK structure in *dark blue*. Mg^2+^ is omitted in all panels for clarity. DMAPP, dimethylallyl pyrophosphate; FPP, farnesyl pyrophosphate; FSP, farnesyl thiophosphate; GGPP, geranylgeranyl pyrophosphate; GPP, geranyl pyrophosphate; MK, mevalonate kinase; RnMK, *Rattus norvegicus* mevalonate kinase; ScMK, *Saccharomyces cerevisiae* mevalonate kinase.

However, a structural alignment shows that unlike FSP, IPP and DMAPP in the ScMK structures bind precisely over the ATP ribose ring in the RnMK structure (Fig. 8*A*, pale blue). Curiously, Ser108 and Asn104 of RnMK that bind the ribose hydroxyl groups are not conserved among MKs that are known to respond to feedback inhibitors (Fig. 7). In fact, both polar and nonpolar residues occupy these positions across different MK homologs, suggesting an evolutionary ambivalence toward the polarity of these positions that is further reflected in the dual binding of polar ATP and nonpolar inhibitors at the same site. For ScMK in particular, Ser108 and Asn104 of RnMK are replaced by Ala114 and Phe110, respectively, converting a polar environment in RnMK into a nonpolar surface in ScMK. Interestingly, the side chain of Phe110 in ScMK fills the same region that was occupied by the backbone carbonyl of the corresponding Asn104 in RnMK that binds ribose (Fig. 8*A*). This difference may suggest a possible strategy for ScMK to switch between binding either ATP or inhibitors, where Phe110 of mobile loop two might switch its conformation to orient either its backbone carbonyl to face the polar ribose of ATP, as in the structure of ATP-bound RnMK, or its nonpolar side chain to face the nonpolar chain of the inhibitor. Such a change in conformation would also be consistent with the flexibility of Phe110 and the loop to which it belongs, as described above. Corroborating evidence of this idea awaits structural information on ATP binding in ScMK.

The next-larger inhibitor, GPP, adds five more carbon atoms in an approximately linear fashion (Figs. 4*C*, 6*B* and 8*B*) that extend past the ribose of ATP in the ATP-bound RnMK structure. As a result, these additional GPP carbons do not occupy the precise location as the adenine ring of ATP, but rather a hydrophobic space just below adenine. Here, adenine in the ATP-bound RnMK structure sits in a primarily nonpolar region comprised of Leu53, Val56, Val133, and Tyr149 (RnMK numbering), the latter forming stacking interactions with the aromatic adenine ring (Fig. 8*D*, green). The only polar interactions between adenine and RnMK are hydrogen bonds between the exocyclic NH_2_ group of adenine and Asn55 and Ser135 (Fig. 8, *A*–*D*), which is strictly conserved (Fig. 7). But because the additional five carbons of GPP lie below the adenine ring’s position, the inhibitor avoids meeting the polar environment of Asn55 and Ser135. In fact, the corresponding residues of ScMK—Asp56 and Ser138, respectively—point away from the ATP-binding site in all ScMK structures presented here (Fig. 8*A*, pale blue). The additional five carbons of GPP instead occupy the deepened level of the hydrophobic pocket described earlier, which is formed by Phe54, Phe110, Ala114, Leu136, Ile142, Ile152, Leu156, and Ile194 (Fig. 6*B*). Further, as Tyr149 in the ATP-bound RnMK structure forms stacking interactions with the adenine ring, not only would Tyr149 clash with GPP binding in ScMK, Tyr149 of RnMK is replaced by the smaller Ile152 in ScMK, which is part of the deepened hydrophobic pocket (Figs. 7 and 8*D*). Inversely, Phe54 in the hydrophobic pocket of ScMK would otherwise clash with the adenine ring of ATP from the RnMK structure, but in RnMK this residue is replaced by Leu53 (Fig. 7). To summarize, differences in ATP and inhibitor binding are not only explained by amino acid differences between RnMK and ScMK, but also by two key strategies: a shift in the positioning of the bound ligand such that nonpolar inhibitors avoid the few polar regions that interact with ATP, as well as changes in side-chain and loop conformations to accommodate the disparate ligands and avoid clashes.

Continuing through the inhibitor-bound ScMK structures presented here, the FPP-bound structure further illustrates these ideas on ligand binding. FPP contains an additional five carbons compared to GPP that must also be accommodated by the enzyme. The aforementioned hydrophobic wall is an obstruction that prevents the longer carbon chain from continuing to extend in a linear fashion, forcing the FPP chain to make a ∼90° turn (Figs. 4*D* and 6*C*). Starting from the carbon chain of GPP that was located below the adenine of ATP in the RnMK structure, the five additional carbons of FPP then curl further away from the adenine position, again avoiding the polar residues that hydrogen bond with adenine (Fig. 8*C*). Instead, the additional FPP carbons interact with hydrophobic Ile57 and Phe59 (Fig. 6*C*). Interestingly, the alkyl chain of FSP from the FSP-bound RnMK structure also makes a ∼90° turn in a similar direction. However, unlike FPP that binds in a lower position than adenine, as described above FSP binds in a position raised higher than the ribose of ATP and the inhibitors reported here, which causes the FSP tail to directly overlap with the adenine of ATP. This difference causes FSP to reach into the polar environment of Asn55 and Ser135, which may seem unfavorable. However, the FSP tail is tilted away from Ser135, and Asn55 is rotated away from FSP, preventing unfavorable interactions (Fig. 8, *B*–*D*, pink). These differences further illustrate how shifts in ligand positioning as well as changes in side-chain orientation represent two strategies that allow for the same site to bind both ATP and nonpolar inhibitors.

Finally, the terminal carbon atoms of the longest inhibitor, GGPP, curl the alkyl chain an additional ∼90° (Figs. 4*E*, 6*D* and 8, *C* and *D*). The direction of this turn brings the alkyl chain even farther from the polar environment that binds the NH_2_ group of the ATP adenine ring and into a nonpolar wedge described above that is composed of the other carbon atoms of GGPP, Ile57, and Phe110 in a newly adopted conformation (Fig. 6*D*). As mentioned, Phe110 is part of a flexible loop (Ser107–His111) that changes conformation depending on the length of the inhibitor (Fig. S3). The composition of this loop is not strictly conserved in RnMK (Fig. 7), yet it adopts a position that is similar to the apo, IPP-, DMAPP-, and GPP-bound ScMK structures. Therefore, these structures further demonstrate that this loop is variable, flexible, and changes its conformation to accommodate ligands of different sizes.

Intriguingly, although many eukaryotic MK homologs are subject to feedback inhibition, MKs from multiple archaeal species have been found to be resistant to inhibition, including MKs from *Methanosarcina mazei* (MmMK) (27), *Methanococcoides burtonii* (MbMK) (32), *Methanosaeta concilii* (49), and *Methanocella paludicola* (49). Of these, crystal structures are available for MmMK (42) and MbMK (32), and comparisons with our new inhibitor-bound ScMK structures may help to explain how different MK architectures can somehow all bind the ATP substrate while some MKs also tightly bind inhibitors in the same site while other MKs do not bind inhibitors at all.

There are as yet no structures that depict ligands bound in the ATP-binding site of archaeal MK for direct comparisons. However, one striking overall difference between archaeal and eukaryotic MK structures is that the N-terminal, ATP-binding domain of archaeal MK adopts a different topology compared to ScMK. In archaeal MK, a short, 5-residue loop (Gly53–Glu57 in MmMK) connects an extended β-sheet of the N-terminal domain to an α-helix near the ATP-binding site (Fig. 9*A*, red). However, in eukaryotic MKs the same β-sheet and α-helix are connected instead by a nearly 50-residue insertion (Lys62–His111 in ScMK) that forms a large lobe composed of three α-helices followed by the flexible ATP/inhibitor-binding loop two detailed above (Ser107–His111) that changes conformation depending on the ligand bound (Fig. 9*B*, dark blue). Not only is this entire lobe absent in archaeal MK, in the archaeal MK structures determined so far the subsequent α-helix lies too close to the inhibitor-binding site in ScMK and would cause a steric clash that obstructs inhibitor binding (Fig. 9*D*). Furthermore, a bulky tyrosine residue of this helix that is conserved only among archaeal MK (Tyr61 in both MmMK and MbMK numbering) protrudes directly into the ATP/inhibitor binding site of ScMK (Fig. 9*D*), which has Ala114 instead. Therefore, it is possible that resistance to inhibition in archaeal MKs is at least partly due to an absence of the ∼50-residue ATP/inhibitor-binding lobe and a difference in positioning of the α-helix that contains the protruding Tyr61, which may prevent inhibitor binding while still allowing ATP to bind. Corroboration of this idea awaits structural characterization of ATP-bound archaeal MK to enable direct comparisons of ligand-bound states.

**Figure 9:**
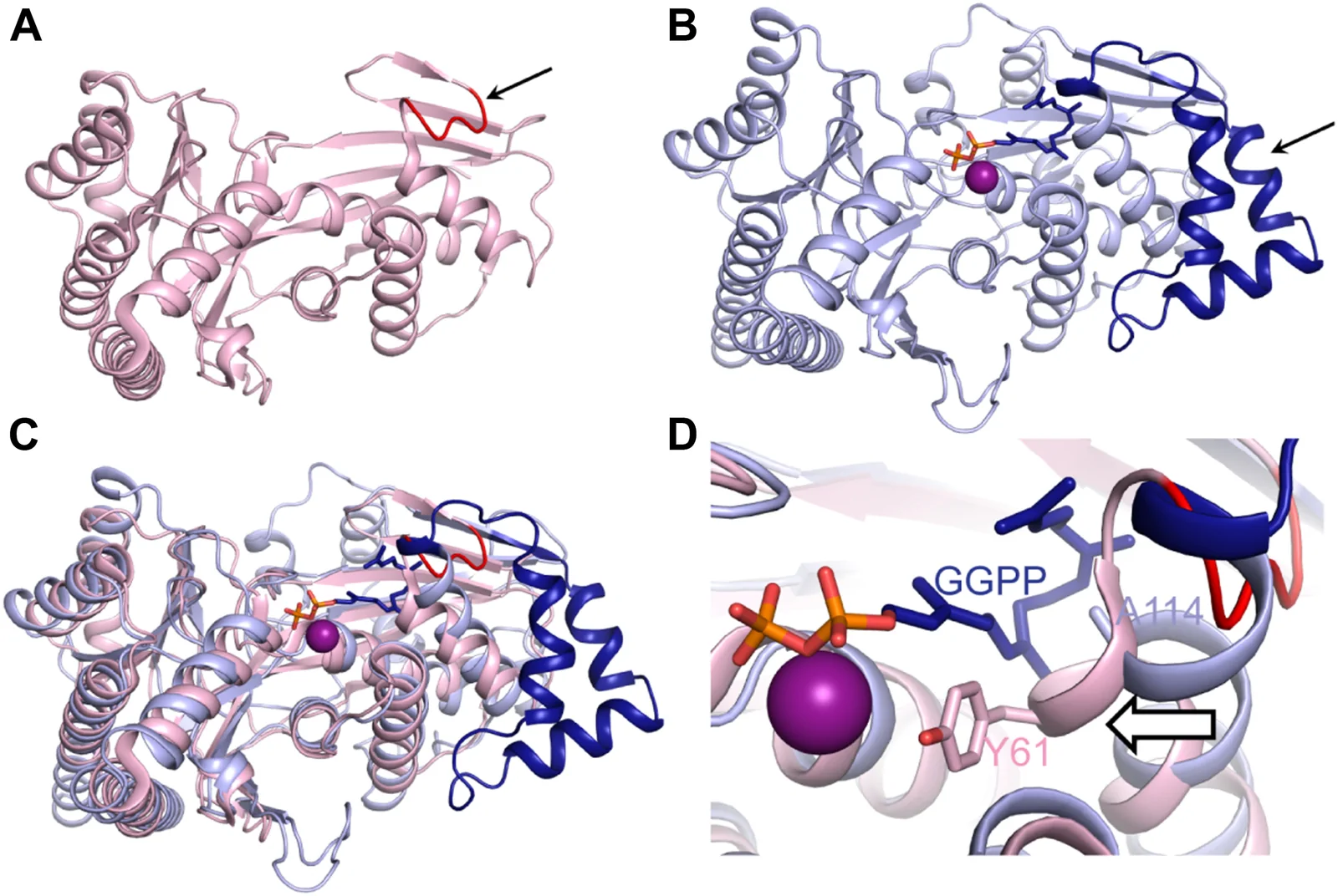
**Structural comparison of ScMK and inhibition-resistant archaeal MmMK** (42)**.***A*, structure of one MmMK monomer (*pink*), with a 5-residue loop near the ATP-binding site shown in *red* and indicated by an arrow (PDB entry 6MDE). *B*, structure of one ScMK monomer (*light blue*) from the structure of ScMK bound with GGPP (*dark blue sticks*), with a ∼50-residue insertion shown in *dark blue* and indicated by an *arrow*. Mg^2+^ shown as a *violet sphere*. *C*, superposition of *A* and *B*. *D*, *Zoom*-*in of C*, highlighting a shift in the position of a helix (*block arrow*) that contains either A114 in ScMK or Y61 in MmMK. MmMK, *Methanosarcina mazei* mevalonate kinase; ScMK, *Saccharomyces cerevisiae* mevalonate kinase.

The suite of inhibitor-bound ScMK structures presented here provides new structural depictions of MK feedback inhibition, a key regulatory mechanism of the mevalonate pathway. In particular, this work uncovers the structural strategies that allow the ATP binding site of MK to also accommodate inhibitors of varying sizes, with both short and long hydrocarbon chains. Here, altered ligand positioning together with side chain rearrangements and small loop movements’ direct nonpolar inhibitor chains away from the few polar groups that otherwise bind ATP, while also exposing hydrophobic regions for favorable binding. In all, these structures could prove invaluable for enzyme engineering efforts to attenuate MK feedback inhibition in engineered microbes that use the mevalonate pathway for industrial isoprenoid production. Given the medical importance of the mevalonate pathway, our new structural insight into MK inhibition also has the potential to drive the development of new drugs that target MK in both humans and in pathogenic microorganisms.

## Experimental procedures

### Protein expression and purification

A codon-optimized gene encoding WT ScMK (Integrated DNA Technologies) was cloned by Gibson assembly (50) into the plasmid pSKB3, a modified pET28b vector with a tobacco etch virus protease-cleavable N-terminal hexahistidine tag and a kanamycin resistance cassette. The plasmid was transformed into *Escherichia coli* DH5α cells, and its gene sequence was confirmed (Quintara Biosciences) before it was transformed into BL21(DE3) cells for protein expression.

Cells were grown in lysogeny broth supplemented with kanamycin (50 μg/ml) at 37 °C until the optical density at 600 nm reached ∼0.8. Expression was induced with 0.5 mM isopropyl β-d-thiogalactopyranoside (IPTG) and continued at 20 °C overnight. Cells were harvested by centrifugation at 5000*g* for 15 min at 4 °C, flash-frozen in liquid nitrogen, and stored at −20 °C.

Cells were thawed and resuspended in lysis buffer (50 mM Tris-HCl pH 7.8, 300 mM NaCl, 10 mM imidazole, 2 mM MgCl_2_) with 0.5 mM PMSF and 0.05 U/μl of Pierce universal nuclease (Thermo Fisher Scientific). Cell lysis was performed at 4 °C by three rounds of sonication at 30 to 35% amplitude, with each round consisting of 2 s pulses and 5 s rests for 9 min. The lysate was clarified by centrifugation at maximum speed (∼30,000*g*) for 60 min at 4 °C. ScMK was purified using Ni-NTA affinity chromatography. The crude supernatant was applied to the Ni-NTA column and mixed at 4 °C for 30 to 60 min. The column was then washed with at least ten column volumes each of lysis buffer followed by lysis buffer with 40 mM imidazole. ScMK was eluted in fractions using lysis buffer with 250 mM imidazole. Protein purity was assessed by sodium dodecyl sulfate (SDS)-PAGE, and fractions containing the greatest purity were combined and dialyzed at 4 °C against storage buffer (50 mM Tris-HCl pH 7.8, 2 mM MgCl_2_). Purified ScMK was concentrated to approximately 20 mg/ml as measured by the absorbance at 280 nm using a calculated extinction coefficient of 33,160 M^−1^ cm^−1^. Final protein concentration was quantified using the Bradford assay (51), and aliquots were flash-frozen in liquid nitrogen before storage at −80 °C.

### Inhibition kinetics

Steady-state competitive inhibition kinetics with respect to ATP were performed using a pyruvate kinase/lactate dehydrogenase assay where ScMK activity was coupled to NADH consumption, which was monitored using a SpectroStar Nano UV/visible microplate reader (BMG Labtech). Each reaction contained 100 mM Tris-HCl pH 7.8, 10 mM MgCl_2_, 10 mM NADH, 35 mM phosphoenolpyruvate (PEP), 0.5 U/μl pyruvate kinase/lactate dehydrogenase (Sigma-Aldrich), 8.3 ng/μl purified ScMK, and 0 to 1.0 mM ATP. Reactions with inhibitors also contained 0 to 500 μM IPP, 0 to 500 μM DMAPP, 0 to 7.5 μM GPP, 0 to 5.0 μM FPP, or 0 to 25 μM GGPP (Isoprenoids, LC), with at least three different concentrations tested for each inhibitor. Reactions were initiated by the addition of 0.5 mM (*R*)-mevalonate (Sigma-Aldrich), and the absorbance at 340 nm was monitored to determine initial rates of NADH consumption using an extinction coefficient of 6220 M^−1^ cm^−1^. All experiments were performed in at least triplicate, where each independent experimental replicate contained one full range of ATP concentrations, used fresh reaction preparations, and was run on a separate microplate reaction. Replicates were then compiled, with data for each inhibitor at different concentrations compiled together. Global nonlinear regression analysis in GraphPad Prism 11 was performed by plotting initial rates against ATP concentration and fitting the compiled data to the Michaelis-Menten equation to determine the maximum velocity, *V*_max_, and the apparent Michaelis-Menten constant, *K*_m,app_, which were used to calculate inhibitor dissociation constants, *K*_i_, using the formula *K*_i_ = *K*_m_ [*I*]/(*K*_m,app_–*K*_m_). The turnover number, *k*_cat_, was calculated by dividing *V*_max_ by the molar enzyme concentration. Values reported are the means ± the SEM.

### Protein crystallization

Crystallization conditions for apo ScMK were determined using sparse-matrix screening by sitting-drop vapor diffusion on a Crystal Gryphon robotic liquid handler (Art Robbins Instruments). Initial hits were then optimized by hanging-drop vapor diffusion set up manually. The best condition contained 2 μl of 6 to 10 mg/ml purified ScMK mixed with 1 μl of crystallization solution (100 mM MES pH 6.0 and 900–1100 mM sodium potassium tartrate) over a reservoir of 0.5 ml crystallization solution. Diamond-shaped tetragonal crystals appeared after 1 to 2 days at room temperature and grew to their largest size after an additional 2 to 3 days.

For the apo ScMK structure, crystals were harvested and soaked in a solution that contained a 50-50 mixture of the crystallization solution and a cryoprotectant solution (16% ethylene glycol, 16% glycerol, 18% sucrose, and 4% glucose) for approximately 5 min before flash-freezing in liquid nitrogen. For the inhibitor-bound ScMK structures, apo ScMK crystals were soaked in a solution that contained a 50-50 mixture of the crystallization solution and the cryoprotectant solution plus 5 mM of IPP, DMAPP, GPP, FPP, or GGPP for 30 to 45 min before flash-freezing in liquid nitrogen.

### X-ray data collection, model building, and refinement

X-ray diffraction data were collected at the Advanced Photon Source beamlines 24-ID-C and 24-ID-E at 100 K. Data were processed in XDS (52) and iMOSFLM (53), and crystals belonged to the space group *P*4_3_2_1_2 with one protein molecule in the asymmetric unit. A CC_1/2_ value in the highest resolution shell of 0.3 was used as the primary resolution cutoff criterion, though in most structures the detector distance limited the resolution before this CC_1/2_ value was reached. The structure of apo ScMK was solved by molecular replacement using Phaser (54) in the Phenix suite (55), with one protein monomer without any ligands bound from the prior structure of RnMK (43), PDB entry 1KVK, as a search model (31% sequence identity). Iterative rounds of model building in Coot (56) with visual inspection of 2*mF*_o_–*d**F*_c_ and *mF*_o_–*d**F*_c_ maps and refinement using phenix.refine (55) were performed.

The structures of inhibitor-bound ScMK were determined by refinement of the refined apo ScMK coordinates against the inhibitor-bound data using phenix.refine (55) and by molecular replacement using Phaser (54) in the Phenix suite (55) using the refined apo ScMK structure as a search model. Iterative rounds of model building in Coot (56) with visual inspection of 2*mF*_o_–*d**F*_c_ and *mF*_o_–*d**F*_c_ maps and refinement using phenix.refine (55) were performed, using simulated annealing in the initial stages. All data sets used the same 5% reflection test set (*R*_free_ flags) as the apo structure, extended to higher resolutions where appropriate. Model quality was assessed using MolProbity (57). Data collection and refinement statistics are given in Table S1.

During model building, three additional regions of positive *mF*_o_–*d**F*_c_ difference electron density peaks were found side-by-side in the active sites of all ScMK structures, including in the apo structure (Fig. S5). The first of these three peak clusters corresponds to the mevalonate-binding site near His19, yet these crystals were not soaked with mevalonate. The protein samples were also unlikely to have encountered mevalonate during expression or purification, as the mevalonate pathway is absent in the *E. coli* expression host. Therefore, modeling of similarly sized organic molecules present in the storage buffer (*i.e.*, Tris), the crystallization solution (*i.e.*, MES or tartrate), or the cryoprotectant solution (*i.e.*, glycerol, ethylene glycol, or glucose) was pursued that could satisfy any of these three electron density peaks across all six structures, which varied somewhat in size and intensity across the six structures (Fig. S5). None of these compounds fit the first peak region; therefore, we left this peak unmodeled in all structures. Tartrate, which was present at ∼1 M concentration in the crystallization solution, fit well into the electron density maps of the second peak in DMAPP-, GPP-, FPP-, and GGPP-bound structures, with only very small negative *mF*_o_–*d**F*_c_ difference density peaks, and so tartrate is present in the final models of these structures. However, refining tartrate in the apo and IPP-bound structures gave strong negative *mF*_o_–*d**F*_c_ difference density peaks (Fig. S5*B*) and no other ligands fit well in this peak, so this region was left unoccupied in these two structures. Finally, the third peak, located within hydrogen-bonding distance of the pyrophosphate head group of the inhibitors, varied in size and clarity across all structures, from a single large electron density peak in the GPP- and GGPP-bound structures to a discontinuous stream of weaker peaks in the other structures. Glucose, used as a cryoprotectant, in its β-d-glucopyranose form fit relatively well into the density maps of the GPP- and GGPP-bound structures (Fig. S5, *D* and *F*), with only small negative *mF*_o_–*d**F*_c_ difference density peaks, and so glucose is present in the final models of these two structures. However, refinement of glucose into the other structures yielded large negative *mF*_o_–*d**F*_c_ difference density peaks and no other ligands fit well in this peak, so this region was left unoccupied in the apo, IPP-, DMAPP-, and FPP-bound structures. Ligand binding in these peaks is unlikely to have affected inhibitor binding, as the phosphate groups of the inhibitors lie in the same positions as previously seen in the ATP- and FSP-bound RnMK structures, and the phosphate-binding region is also unchanged in these structures (Fig. 5). In addition, inhibitors differ from each other and from ATP in their hydrophobic tails, which are in the opposite direction from these density peaks and lie within in a hydrophobic pocket that is distant from these unknown peaks.

## Data availability

Crystal structure coordinates and structure factor data for apo ScMK and ScMK bound with IPP, DMAPP, GPP, FPP, and GGPP are deposited in the PDB with accession codes 36WI, 36WJ, 36WK, 36WL, 36WM, and 36WN, respectively.

## Supporting information

This article contains supporting information.

## Conflict of interest

The authors declare that they have no conflicts of interest with the contents of this article.
