# Supplementary material for: Structural basis of mevalonate pathway regulation by feedback inhibition of mevalonate kinase
Source: J Biol Chem. 2026 Sep 17;302(11):113566. doi: 10.1016/j.jbc.2026.113566 (PMC13683438; doi:10.1016/j.jbc.2026.113566)
Supplement: Supplementary file 1 [file mmc1.pdf]

Supporting Information

Structural basis of mevalonate pathway regulation by feedback inhibition of mevalonate kinase

Rachel L.S. D'Emilia,[1] Kimberly A. McCaskey,[1] Edwin R. Ragwan,[1,2] Jude H. Kim,[1] Vivian Chang,[1] My M. Tang,[1] and Yan Kung[1]*

1. Department of Chemistry, Bryn Mawr College, Bryn Mawr, PA 19010
2. Current address: Department of Chemistry and Biochemistry, Villanova University, Villanova, PA 19085

* Correspondence to: Yan Kung, Department of Chemistry, Bryn Mawr College, 101 N. Merion Ave., Bryn Mawr, PA 19010. E-mail: ykung@brynmawr.edu

Running title: Structural basis of mevalonate kinase feedback inhibition

Contents:
Table S1
Figures S1–S5

Table S1. X-ray data collection and refinement statistics.

	apo ScMK	ScMK + IPP	ScMK + DMAPP	ScMK + GPP	ScMK + FPP	ScMK + GGPP
PDB ID	36WI	36WJ	36WK	36WL	36WM	36WN
**Data collection**						
Beam line	APS, 24-ID-C	APS, 24-ID-C	APS, 24-ID-E	APS, 24-ID-E	APS, 24-ID-E	APS, 24-ID-E
Wavelength (Å)	0.97918	0.97911	0.97918	0.97918	0.97918	0.97918
Space group	$P4_32_12$	$P4_32_12$	$P4_32_12$	$P4_32_12$	$P4_32_12$	$P4_32_12$
Unit cell dimensions						
a, c (Å)	83.45, 262.88	83.97, 262.32	83.63, 263.08	83.45, 262.88	83.85, 262.89	83.48, 262.63
Resolution (Å)	83.45–2.15 (2.21–2.15)	79.97–2.08 (2.14–2.07)	83.63–1.93 (1.98–1.93)	83.59–2.05 (2.12–2.05)	87.67–2.09 (2.16–2.09)	83.48–1.95 (2.00–1.95)
Wilson B (Å^2)	42.53	36.60	37.18	33.24	35.99	25.88
Total reflections	196,216	252,426	312,814	389,913	367,894	1,834,788
Unique reflections	49,876	56,693	70,272	58,995	55,993	68,896
Multiplicity	3.9 (4.0)	4.5 (4.7)	4.5 (4.6)	6.6 (6.9)	6.6 (6.8)	26.6 (27.5)
Completeness (%)	97.5 (99.0)	98.9 (99.1)	99.2 (99.6)	100.0 (100.0)	100.0 (100.0)	100.0 (100.0)
Mean I/σ(I)	5.9 (0.8)	5.6 (0.9)	8.5 (0.8)	12.4 (2.7)	7.8 (1.0)	11.8 (2.2)
R_{merge}	0.131 (2.051)	0.159 (1.903)	0.088 (1.930)	0.088 (0.814)	0.163 (2.219)	0.282 (1.981)
R_{pim}	0.072 (1.122)	0.083 (0.972)	0.045 (0.984)	0.037 (0.330)	0.068 (0.907)	0.055 (0.382)
$CC_{1/2}$	0.995 (0.305)	0.958 (0.431)	0.998 (0.447)	0.997 (0.849)	0.997 (0.495)	0.998 (0.383)
**Refinement**						
R_{work}	0.1982	0.1963	0.1892	0.1779	0.1925	0.2034
R_{free}	0.2236	0.2247	0.2091	0.1978	0.2157	0.2282
r.m.s.d. bonds (Å)	0.007	0.007	0.007	0.007	0.008	0.007
r.m.s.d. angles (°)	0.944	0.890	0.890	0.859	0.979	0.924
Ramachandran analysis						
Favored (%)	97.33	97.33	97.56	98.22	97.33	97.11
Allowed (%)	2.67	2.45	2.22	1.56	2.44	2.67
Outliers (%)	0.00	0.22	0.22	0.22	0.22	0.22
MolProbity Clashscore	4.35	4.29	2.18	2.15	2.74	3.46

* Statistics for highest resolution shell in parentheses

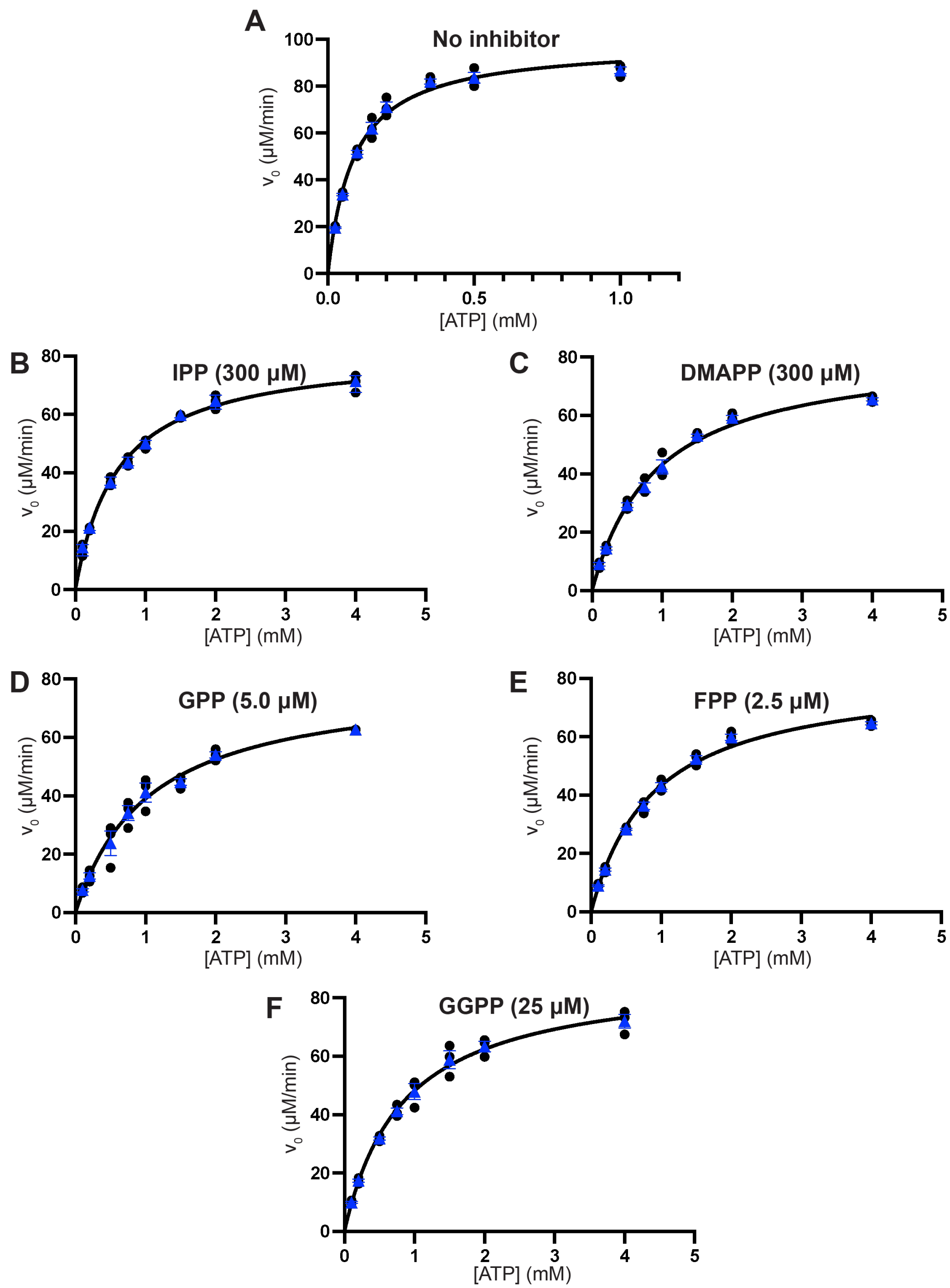

Figure S1. Representative Michaelis-Menten plots for steady-state kinetics experiments of ScMK with **(A)** no inhibitor, or in the presence of various concentrations of **(B)** IPP, **(C)** DMAPP, **(D)** GPP, **(E)** FPP, and **(F)** GGPP. Experiments were performed in at least triplicate, with replicates shown in black circles and averages in blue triangles with blue error bars representing the standard error of the mean (SEM). Data were fit to the Michaelis-Menten equation in GraphPad Prism 11. To calculate K_i values, compiled data from at least 3 different inhibitor concentrations were fit globally.

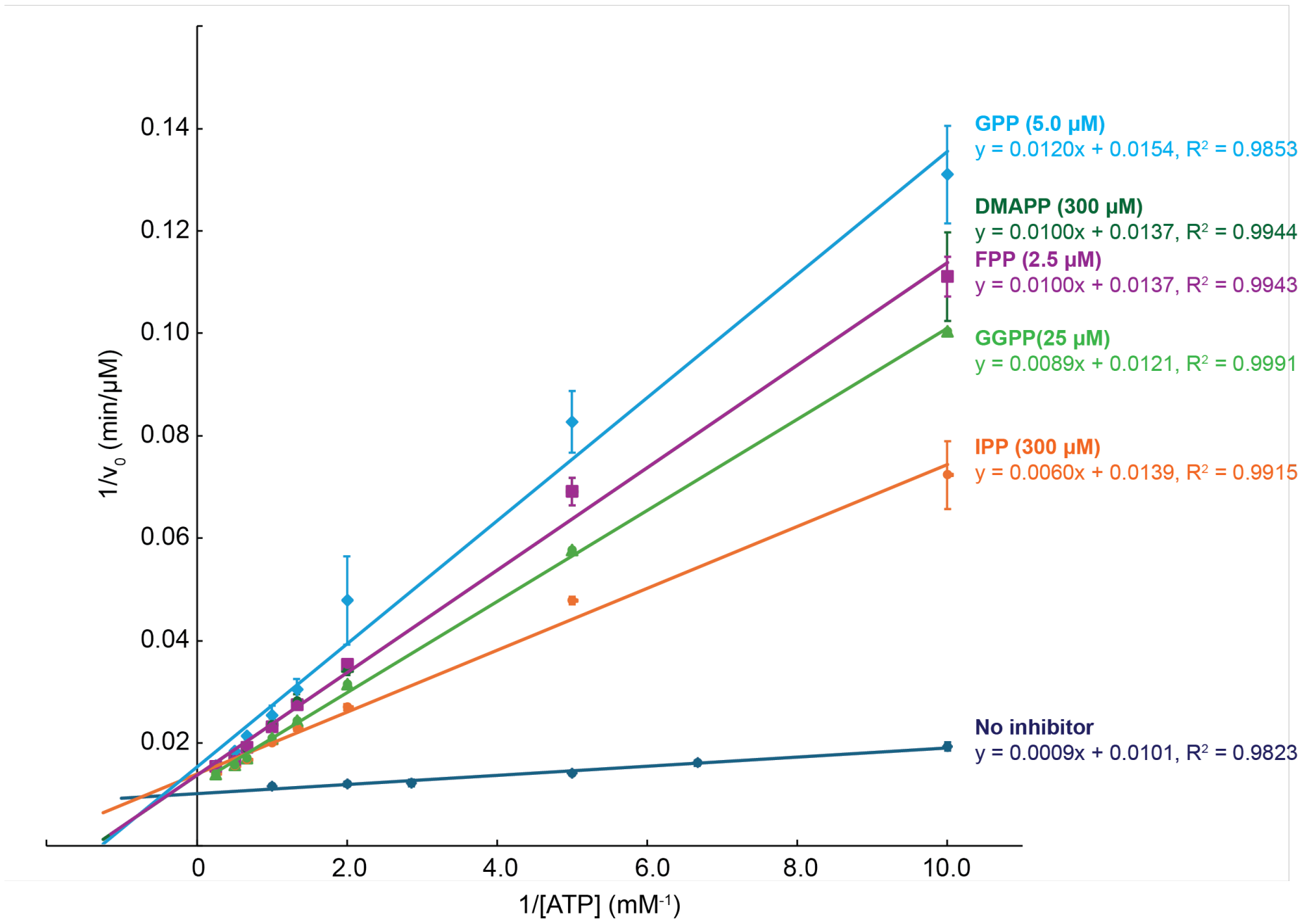

Figure S2. Double-reciprocal plots of the data shown in Figure S1 from representative steady-state kinetics experiments of ScMK with no inhibitor (dark blue) or in the presence of various concentrations of IPP (orange), DMAPP (dark green), GPP (light blue), FPP (violet), and GGPP (light green). Linear fits are shown as straight lines; note that lines for DMAPP and FPP experiments are virtually superimposed. Experiments were performed in at least triplicate, with error bars representing the standard error of the mean (SEM).

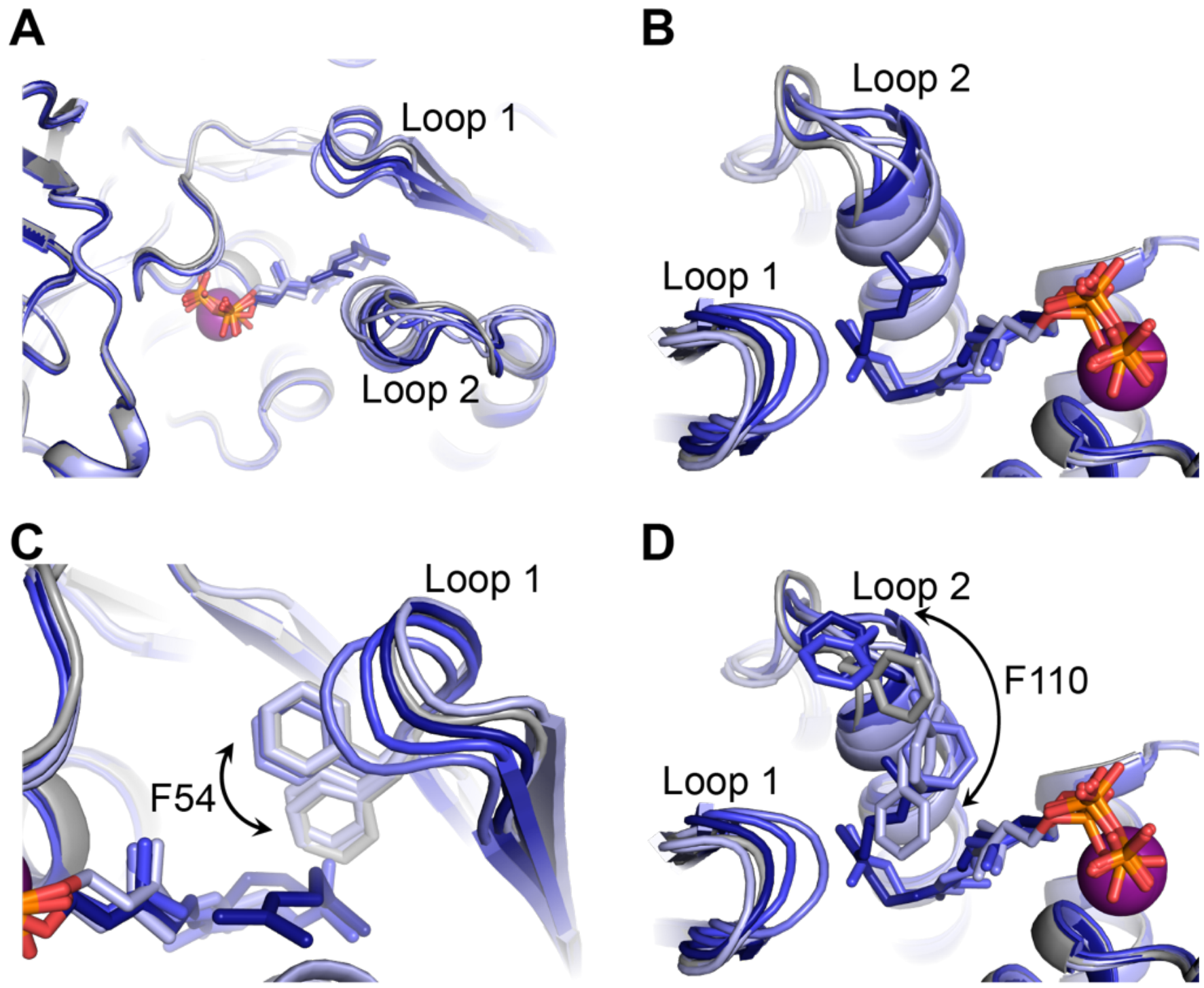

Figure S3. Two flexible loops near the inhibitor-binding site in structures of apo and inhibitor-bound ScMK. Loop 1: Phe54–Phe59, and loop 2: Ser107–His111. Apo ScMK structure in gray, and ScMK structures bound with IPP, DMAPP, GPP, FPP, and GGPP in pale blue, pale blue, light blue, medium blue, and dark blue, respectively, with ligands in sticks and Mg^{2+} as a violet sphere. **(A)** Wide view of both loops near the inhibitor-binding site, **(B)** reverse view, **(C)** zoom-in on loop 1 from a similar orientation as (A), with Phe54 from loop 1 of all structures shown in sticks, and **(D)** Same as (B), with Phe110 from loop 2 of all structures shown in sticks.

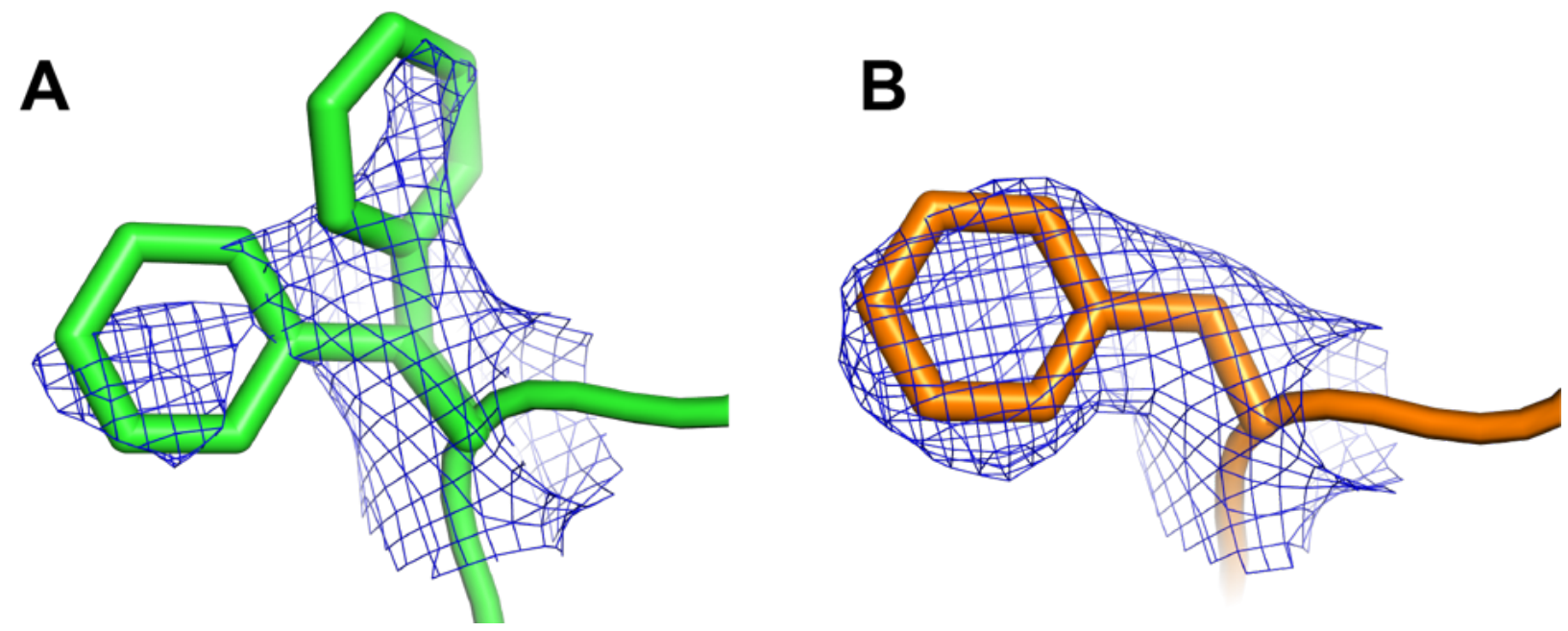

Figure S4. Electron density for Phe54 in **(A)** the apo ScMK structure (green), with two alternate conformations in relatively disordered electron density, and **(B)** the GPP-bound ScMK structure (orange), with one clear conformation. $2mF_o–DF_c$ electron density maps are shown in blue mesh contoured at 1.0 σ.

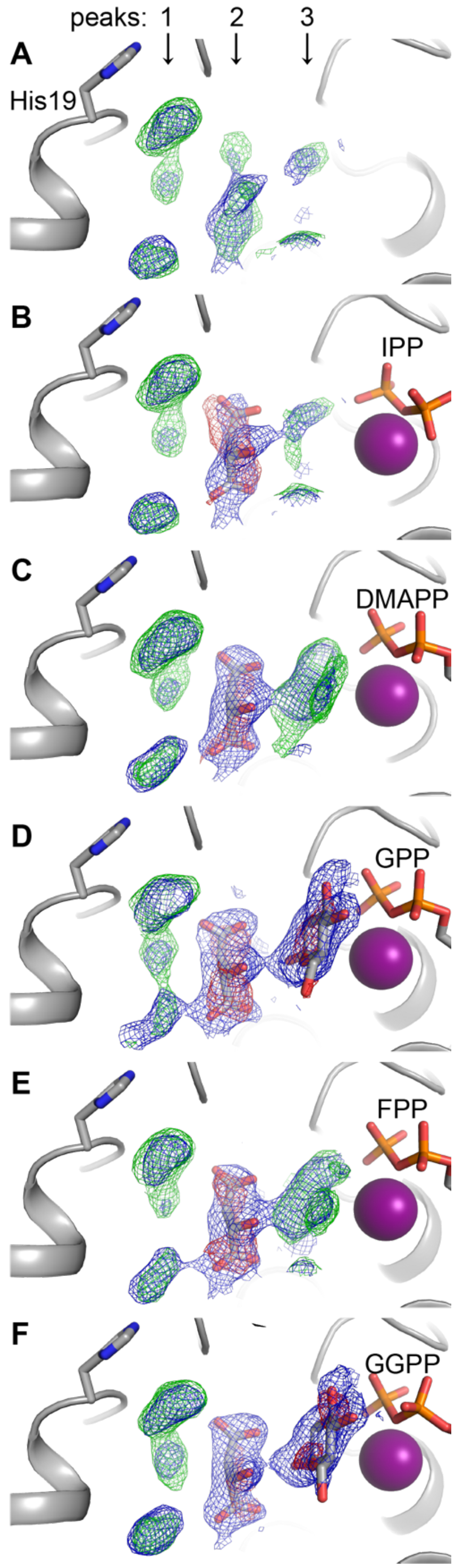

Figure S5. Three electron density peaks in the active sites of ScMK crystal structures (gray) with **(A)** no inhibitor, **(B)** IPP, **(C)** DMAPP, **(D)** GPP, **(E)** FPP, and **(F)** GGPP (inhibitors in sticks, Mg^{2+} as a violet sphere). Peak 1 was left unmodeled in all structures. The final models of DMAPP-, GPP-, FPP-, and GGPP-bound ScMK have tartrate in peak 2, but this peak was left unmodeled in apo and IPP-bound ScMK. The final models of GPP- and GGPP-bound ScMK have β-D-glucopyranose in peak 3, but this peak was left unmodeled in all other structures. $2mF_o$–DF_c maps in blue mesh (1.0 σ) and mF_o–DF_c maps in green (3.0 σ) and red (–3.0 σ) mesh.
